# High‐gamma power changes after cognitive intervention: preliminary results from twenty‐one senior adult subjects

**DOI:** 10.1002/brb3.427

**Published:** 2016-01-30

**Authors:** Yoritaka Akimoto, Takayuki Nozawa, Akitake Kanno, Toshimune Kambara, Mizuki Ihara, Takeshi Ogawa, Takakuni Goto, Yasuyuki Taki, Ryoichi Yokoyama, Yuka Kotozaki, Rui Nouchi, Atsushi Sekiguchi, Hikaru Takeuchi, Carlos Makoto Miyauchi, Motoaki Sugiura, Eiichi Okumura, Takashi Sunda, Toshiyuki Shimizu, Eiji Tozuka, Satoru Hirose, Tatsuyoshi Nanbu, Ryuta Kawashima

**Affiliations:** ^1^ Department of Functional Brain ImagingInstitute of Development, Aging and CancerTohoku UniversitySendai980‐8575Japan; ^2^ Smart Ageing International Research CenterInstitute of Development, Aging and CancerTohoku UniversitySendai980‐8575Japan; ^3^ Japan Society for the Promotion of Science (JSPS)Tokyo102‐8472Japan; ^4^ Division of Developmental Cognitive NeuroscienceInstitute of Development, Aging and CancerTohoku UniversitySendai980‐8575Japan; ^5^ Human and Social Response Research DivisionInternational ResearchInstitute of Disaster ScienceTohoku UniversitySendai980‐8575Japan; ^6^ Division of Medical Neuroimage AnalysisDepartment of Community Medical SupportsTohoku Medical Megabank OrganizationTohoku UniversitySendai980‐8575Japan; ^7^ Department of EpileptologyTohoku University Graduate School of MedicineSendai980‐8575Japan; ^8^ Mobility Services LaboratoryResearch Division 2Nissan Motor Co., Ltd.Kanagawa243‐0123Japan; ^9^ Vehicle Test and Measurement Technology Development DepartmentCAE and Testing Division 1Nissan Motor Co., Ltd.Kanagawa243‐0192Japan; ^10^ Prototype and Test DepartmentResearch Division 2Nissan Motor Co., Ltd.Kanagawa243‐0123Japan; ^11^ Department of Adult Mental HealthNational Institute of Mental HealthNational Center of Neurology and PsychiatryKodaira187‐8553Japan

**Keywords:** Behavioral performance, cognitive intervention, elderly, high‐gamma activity

## Abstract

**Introduction:**

Brain‐imaging techniques have begun to be popular in evaluating the effectiveness of cognitive intervention training. Although gamma activities are rarely used as an index of training effects, they have several characteristics that suggest their potential suitability for this purpose. This pilot study examined whether cognitive training in elderly people affected the high‐gamma activity associated with attentional processing and whether high‐gamma power changes were related to changes in behavioral performance.

**Methods:**

We analyzed (MEG) magnetoencephalography data obtained from 35 healthy elderly subjects (60–75 years old) who had participated in our previous intervention study in which the subjects were randomly assigned to one of the three types of intervention groups: Group V trained in a vehicle with a newly developed onboard cognitive training program, Group P trained with a similar program but on a personal computer, and Group C was trained to solve a crossword puzzle as an active control group. High‐gamma (52–100 Hz) activity during a three‐stimulus visual oddball task was measured before and after training. As a result of exclusion in the MEG data analysis stage, the final sample consisted of five subjects in Group V, nine subjects in Group P, and seven subjects in Group C.

**Results:**

Results showed that high‐gamma activities were differently altered between groups after cognitive intervention. In particular, members of Group V, who showed significant improvements in cognitive function after training, exhibited increased high‐gamma power in the left middle frontal gyrus during top‐down anticipatory target processing. High‐gamma power changes in this region were also associated with changes in behavioral performance.

**Conclusions:**

Our preliminary results suggest the usefulness of high‐gamma activities as an index of the effectiveness of cognitive training in elderly subjects.

## Introduction

Cognitive training improves several cognitive functions, including processing speed, attention, and executive functions, not only in healthy young adults but also in senior adults (Nouchi et al. 2012, 2013). Recently, brain‐imaging techniques have begun to be popular to evaluate the effectiveness of cognitive training in elderly people [for reviews, please see 3]. Several MR (magnetic resonance) imaging studies have examined neural structural and functional changes, which might represent the underlying mechanism of improvements in cognitive performance (Takeuchi et al. 2010; Chapman et al. 2013). Gamma oscillatory activity, which is measured by EEG (electroencephalography) or MEG (magnetoencephalography) and has been observed during various perceptual and cognitive processes (Fries 2009), has only rarely been used to evaluate the effectiveness of cognitive training. However, gamma activities have several characteristics that are suitable for this purpose. Practically, EEG and MEG are safely applicable to children, patients, and elderly people who are often targets for cognitive intervention, but are less suitable for MRI scanning. Theoretically, gamma‐band event‐related synchronization is considered a signature of local circuit operations (Buzsáki and Wang 2012). Gamma activities show more specific timing and localization than lower frequencies, such as, theta (Crone et al. 2006), which has been shown to be useful for evaluating training effects (Anguera et al. 2013). Gamma activities also show considerable individual differences and intraindividual reliabilities (Keil et al. 2003; Fründ et al. 2007). In addition, previous studies have revealed that individual differences in gamma activities predict individual variations in behavioral performance (Hoogenboom et al. 2010; Akimoto et al. 2013, 2014). For example, our previous study revealed that those who exhibit higher high‐gamma (52–100 Hz) power in the left MFG (middle frontal gyrus), the left IPS (intraparietal sulcus), and the left thalamus show better performance during a three‐stimulus visual oddball task (Akimoto et al. 2014). Furthermore, a few reports have shown that cognitive training affects gamma activity during training‐related perceptual processes (Shahin et al. 2008; Popov et al. 2012). Shahin et al. (2008) have measured gamma‐band activity in 4‐ and 5‐year‐olds before starting music lessons and 1 year later. They found that the children receiving music training exhibited increased gamma activity during the passive listening of sounds. Popov et al. (2012) conducted 4 weeks of cognitive training in patients with schizophrenia, and they have found that cognitive training for auditory‐verbal discrimination increased gamma activity during the passive listening of sound. However, it remains unclear whether cognitive training affects gamma activity in elderly subjects.

Our aim in this pilot study was to evaluate the usefulness of high‐gamma activities as an index of the efficacy of cognitive training in elderly subjects. For this purpose, we investigated whether cognitive training in elderly people affected the high‐gamma activity associated with attentional processing and whether high‐gamma power changes were related to changes in behavioral performance. Recently, we conducted an intervention study (Nozawa et al. 2015) to investigate the effects of different types of cognitive training on cognitive function and driving safety: Group V trained in a vehicle with a newly developed onboard cognitive training program, Group P trained with a similar program but on a personal computer, and Group C was trained to solve a crossword puzzle as an active control group. After the 8‐week intervention, Group V showed significant improvement in the processing speed (*P *=* *0.048) and working memory (*P *=* *0.048) composites. Additionally, marginally significant improvements were observed in Group V in the executive function (*P *=* *0.076) and cognitive impairment (COGSTAT) (*P *=* *0.076) composites, and in Group C in the working memory (*P *=* *0.092) and COGSTAT (*P *=* *0.078) composites. In this study, we analyzed the MEG data that were obtained when these senior adult subjects conducted a three‐stimulus visual oddball task before and after the intervention periods. On the basis of previous meta‐analysis results indicating that reliable near‐transfer effects were obtained from executive function and working memory training in elderly subjects (Karbach and Verhaeghen 2014), we expected improvement in behavioral performance, and more importantly an alteration in high‐gamma activities after cognitive intervention in Group V. This is because oddball processing has been associated with executive function and working memory as well as other attentional processes (Huettel and McCarthy 2004; Polich 2007; West et al. 2010), which were targeted in the cognitive intervention in Groups V and P. Our specific hypotheses were first, that Group V would exhibit the highest high‐gamma power increase in the left MFG, the left IPS, and the left thalamus in oddball processing (but not in the processing of standard stimuli) after the intervention, because Group V showed the best improvements in cognitive function after training. And, from the point of view of near‐ and far‐transfer effects, Group P could achieve greater improvement than Group V as both the oddball task and the training in Group P were conducted in a common setting (i.e., personal computer). However, we expected a weaker effect in Group P than in Group V, given that no significant improvement was observed in Group P in the neuropsychological test battery evaluation. We had no specific expectations about Group C, whose cognitive intervention did not relate to the current task but showed marginally significant improvement in the working memory and COGSTAT composites. A previous meta‐analysis found that significant but small far‐transfer effects in working memory training in elderly subjects (Karbach and Verhaeghen 2014). Among the target brain regions, we expected to observe increased high‐gamma power most likely in the left MFG, which is an important region for executive control and top‐down anticipatory attention (Liang and Wang 2003; Aron et al. 2004). It has been repeatedly shown that elderly people exhibit greater recruitment of prefrontal regions and reduced activation of posterior regions in various cognitive tasks (O'Connell et al. 2012; Van Dinteren et al. 2014). Therefore, we assumed that our elderly subjects had relied most heavily on prefrontal regions during training, and thus, the training effect would be most evident in the prefrontal regions. Second, we predicted that high‐gamma activity changes that occurred during target processing would be associated with changes in behavioral performance regardless of group. In other words, we expected that high‐gamma power was sensitive to changes in behavioral performance occurring for any reason.

## Methods

### Subjects

Thirty‐seven healthy senior adults (60–75 years old) participated in this study. They were the same subjects as those in our previous intervention study (Nozawa et al. 2015). The subjects were randomly assigned to one of the three groups (Group V, P, or C) by a computerized random draw with a balanced sex ratio among the groups (Nozawa et al. 2015). However, the balanced number of subjects and sex ratio among the groups were eventually lost in the final sample, as described below. Although the data for the preintervention period were reported in our previous MEG study (Akimoto et al. 2014), the data for the postintervention period have not yet been reported. We also noted that some of the subjects who were included in our previous MEG study were not included in this study because their interventions were disrupted by the 2011 Japan earthquake. In order to exclude those with potential dementia, the exclusion criterion of a Mini‐Mental State Examination score <25 was employed (Folstein et al. 1975). None of the participants was excluded based on this criterion. Those who had dental metal in their mouth were not excluded from the study, but some were eventually excluded from the analysis due to high magnetic noise. One participant withdrew consent before the group allocation, and one participant in Group P dropped out during the intervention period. We excluded three subjects who showed low accuracy in the target condition (two standard deviations below the mean), two subjects who had extremely large reaction times (two standard deviations above the mean), four subjects who exhibited brain atrophy, and five subjects who had trials available for less than the half of the conditions in either of the pre/postintervention periods due to a large amount of magnetic noise. As a result, five subjects in group V, nine subjects in group P, and seven subjects in group C were included in the final analysis. Baseline characteristics of the subjects in the final total sample are presented in Table 1. They were not significantly different among training groups. Written informed consent was obtained from each participant. The Ethics Committee of the Tohoku University Graduate School of Medicine approved the protocol of this study. The study was conducted in accordance with the Declaration of Helsinki.

**Table 1:** Baseline characteristics of the subjects in the final sample

	Group C *n* = 7 (2 F/5 M)		Group P *n* = 9 (4 F/5 M)		Group V *n* = 5 (1 F/4 M)		Main effect of group	
	Mean	SD	Mean	SD	Mean	SD	*F*‐value	*P*‐value
Age (year)	67.31	4.97	68.71	5.96	67.34	5.59	0.160	0.854
Education (year)	14.86	1.57	13.44	2.40	13.60	2.19	0.972	0.397
MMSE base (score)	28.71	1.11	28.00	1.80	28.00	1.23	0.543	0.590

F, female; M, male; SD, standard deviation; Education, number of years of education completed; MMSE, Mini‐Mental State Examination.

The base score indicates the preintervention test score. The main effect of group was tested with one‐way analysis of variance (ANOVA).

### Cognitive interventions

Group V trained in a vehicle with an on‐board cognitive training program. In the training task, color stimuli were presented rhythmically and randomly by five LEDs placed around the driver's seat (four lights: top‐right, bottom‐right, top‐left, and bottom‐left were approximately equidistant from the center of the driver's view; an additional LED was located beside the side mirror on the passenger side). Participants were instructed to turn the steering wheel when two lights of the same color (green or blue, but not yellow) were presented on the same side (left or right). Yellow lights were distractors, so participants were expected to suppress their response even if two yellow lights were presented on the same side. Participants were also instructed to press the brake pedal when red lights were presented at random times by any of the five LEDs. Training comprised both an immediate response version and a delayed response (i.e., n‐back) version. The system provided adaptive training by dynamically adjusting tempo according to subjects' successive correct or incorrect responses. In sum, the training focused on processing speed, executive control, visual processing, divided attention, and working memory. Group P trained with a similar program but on a personal computer. This condition was intended to examine the hypothesized advantage of an on‐board training program over training on a PC in terms of transfer of training effects to cognitive functions and driving safety. Group C was trained to solve a crossword puzzle, and thus served as an active control group. Participants visited our laboratory 24 times (3 days a week for 8 weeks) and completed 20 min of training each time. Before and after the training intervention period, participants completed a neuropsychological test battery including the Block Design subtest of the Wechsler Adult Intelligence Scale III (Wechsler 1997), Frontal Assessment Battery at bedside (Dubois et al. 2000), Word Fluency Test (Lezak 1995), Trail Making Test (Reitan 1958), Symbol‐Digit Modalities Test (Smith 1984), Spatial Span subtest of the Wechsler Memory Scale‐Revised (Wechsler 1987), Benton visual retention test (Benton 1963), Rey‐Osterrieth Complex Figure Test (Shin et al. 2006), Rey Auditory‐verbal learning test (Schmidt 1996), and Judgment of Line Orientation (Benton et al. 1983). Cognitive function was evaluated in four domains (processing speed, executive function, working memory, and cognitive impairment; COGSTAT) by calculating a composite measure across multiple neuropsychological tests. For more detail, see (Nozawa et al. 2015).

### Three‐stimulus oddball task

Before and after the training intervention period, participants completed a three‐stimulus visual oddball task, which was the same as that used in our previous study (Akimoto et al. 2013, 2014), with MEG measurements. In this task, target, nontarget, and standard stimuli were presented with an appearance rate of 10%, 10%, and 80%, respectively. The standard stimuli were blue elliptical shapes, the height and width of which subtended visual angles of 4.6° and 4.1°, respectively. The target stimuli were larger blue elliptical shapes, the height and width of which subtended visual angles of 5.6° and 4.6°, respectively. The infrequent nontarget stimuli were large blue rectangles, the height and width of which subtended visual angles of 6.4° and 5.6°, respectively. Subjects pushed a button with their right index finger only when the target stimulus appeared. Each stimulus was presented for 500 msec. The interstimulus interval was 1500 msec. There were 260 trials in all.

### MEG measurements and analysis

The MEG data were acquired with a whole‐head 200‐channel MEG system (PQA160C, Yokogawa Electric Corporation, Tokyo, Japan) with a sample rate of 2000 Hz and a band‐pass filter of 0.01–500 Hz. The head shape of each participant was digitized with a three‐dimensional digitizer (FastSCAN Cobra, Polhemus, Colchester, VT) and coregistered with individual structural MR images. Structural MR images were acquired with a 3T MR system (Achieva, Philips Healthcare, Best, the Netherlands).

We analyzed the MEG data with a similar procedure as that used in our previous study (Akimoto et al. 2013, 2014). The left MFG (Montreal Neurological Institute coordinates: −25, 24, 48), left IPS (−25, −46, 53), and left thalamus (−5, −26, 8) were defined as ROIs (regions of interest) because their high‐gamma power measures have been previously shown to be significant indicators of attentional ability (Akimoto et al. 2013, 2014). We also noted that these regions did not overlap with the regions showing structural changes after cognitive intervention (Nozawa et al. 2015). The Fieldtrip software package (Oostenveld et al. 2011) was used to conduct an independent component analysis of the MEG data and the typical noise components (e.g., eye blink) were removed by a researcher based on visual inspection. The data were reconstituted from the independent components that remained after the removal of the artifact independent components. We applied the array‐gain‐constraint version of diagonal loading (see the Supplement of (Ueno et al. 2012)) to all of the source reconstructions in this study. The data after preprocessing were band‐pass filtered in the 52–100‐Hz frequency domain. We extracted the brain activities in each ROI with a narrow‐band adaptive beamformer (Dalal et al. 2008). Group level analyses were conducted in the Montreal Neurological Institute space after the individual data were normalized with SPM8 (Wellcome Department of Cognitive Neurology, London, U.K.) with a standard T1 template image. The trials were epoched from 300 msec before stimulus onset to 600 msec after stimulus onset. The trials with incorrect responses or magnetic flux in excess of 2000 fT in any channel were excluded. The number of analyzed trials was nearly equalized among the conditions in order to produce similar noise levels across conditions (Herdman and Cheyne 2009). This was done by randomly selecting the same number of standard trials and nontarget trials for analysis. The average number of analyzed nontarget‐, target‐, and standard‐stimulus trials were 24.2 [standard error of the mean (SE) = 0.46], 23.0 (SE = 0.50), and 24.2 (SE = 0.46), respectively.

We calculated the power changes relative to baseline with a logarithmic conversion with 100‐msec moving windows starting at stimulus onset (0 msec) to 400 msec after stimulus onset. The baseline was the time between −300 and 0 msec of the stimulus onset. Then, the high‐gamma power change (Post – Pre) was computed for each subject. In order to examine the effects of each cognitive intervention, we conducted permutation tests of an ANCOVA (analysis of covariance) for the high‐gamma power changes in each time window in each condition and ROI. The permutation test for ANCOVA was conducted with the “aovp” function of the lmperm package (http://cran.r-project.org/web/packages/lmPerm/index.html) in R version 3.0.1 (http://www.rproject.org/). The high‐gamma power change (Post – Pre) was the dependent variable, and the group was the independent variable. The high‐gamma power of the preperiod, sex, and age were included in the model as covariates. The effects of the intervention on task accuracy {i.e., discriminability index (A') based on signal detection theory (Grier 1971)} and reaction time were analyzed in a similar way with the permutation test for ANCOVA. In addition, we conducted partial correlation analyses between the changes in high‐gamma power in the target condition and the reaction time in all subjects (i.e., collapsing intervention groups) in each time window and ROI with the high‐gamma power of the preperiod, sex, and age as covariates. The statistical threshold was set at *P *<* *0.05 with the false discovery rate method (Benjamini and Hochberg 1995) with controlling the number of time windows and ROIs. If significant effects were found in these analyses, we would also examine partial correlations between change in high‐gamma power and change in the cognitive composites scores, adjusting the high‐gamma power of the preperiod, the cognitive composite scores of the preperiod, sex, and age.

## Results

**Figure 1:**
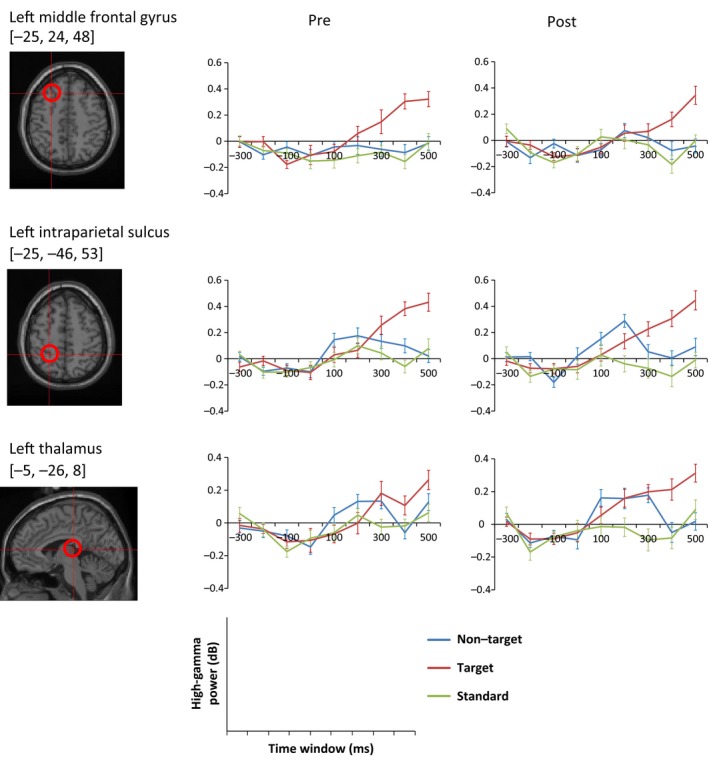
Time course of the high‐gamma power changes averaged for all the subjects in the pre/postintervention periods. The error bars indicate standard errors of the mean.

The behavioral results are presented in Table 2. There was no significant effect of training group either on task accuracy or reaction time (*P *=* *0.723 and *P *=* *0.200, respectively). The time course of the high‐gamma power measurements for all of the subjects are shown in Figure 1, indicating the involvement of all of these regions in top‐down attentional processing specific to the target trials.

**Table 2:** Behavioral performance in each group in each intervention period

	RT				A'			
	Pre		Post		Pre		Post	
Group	Mean	SD	Mean	SD	Mean	SD	Mean	SD
C	504.1	69.7	508.9	46.2	0.976	0.026	0.983	0.009
P	464.3	41.2	510.9	63.1	0.978	0.019	0.983	0.019
V	480.4	68.0	465.5	63.2	0.985	0.012	0.990	0.007

RT, reaction time; A', discriminability index; SD, standard deviation.

**Figure 2:**
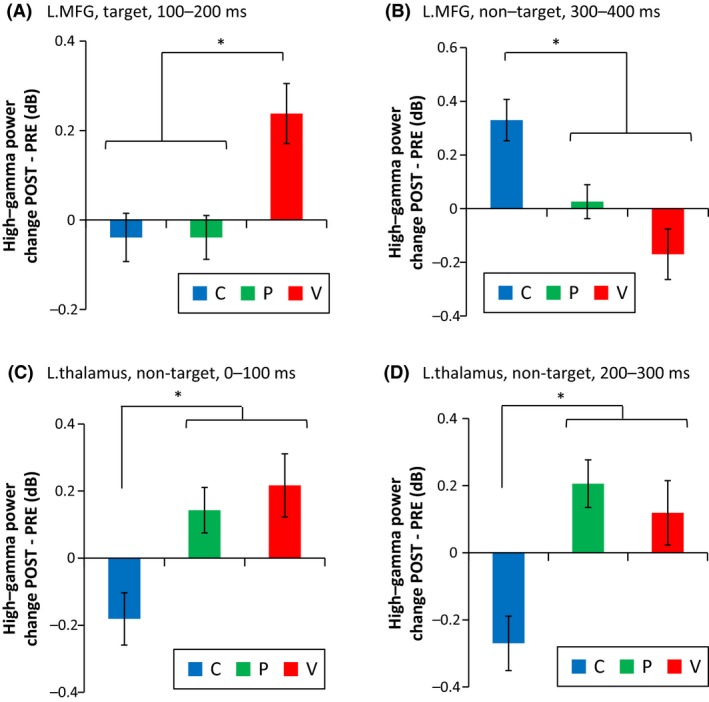
Changes in high‐gamma power (dB) in each training group. (A) High‐gamma power changes in the target condition from 100 to 200 msec in the left MFG. (B) High‐gamma power change in the nontarget condition from 300 to 400 msec in the left MFG. (C) High‐gamma power change in the nontarget condition in the left thalamus from 0 to 100 msec and (D) from 200 to 300 msec. The asterisks indicate significant differences (*P* < 0.05). The number of time windows, the stimulus conditions, and the regions of interests were not corrected. The values were adjusted for age, sex, and pre (baseline) values. The error bars in the graphs indicate the standard error of the mean for the subjects in each group. Multiple comparison correction was not applied.

The ANCOVA permutation tests revealed no significant effects of training group. Without the multiple comparison correction, however, we found effects of training group on the high‐gamma activities in the target condition from 100 to 200 msec in the left MFG, in the nontarget condition from 300 to 400 msec in the left MFG, and in the nontarget condition from 0 to 100 msec and from 200 to 300 msec in the left thalamus (Fig. 2). Consistent with our expectations, the posthoc comparisons revealed that Group V showed the greatest increase in high‐gamma power in the left MFG from 100 to 200 msec in the target condition. In addition, Groups V and P showed increased high‐gamma power compared to Group C (which exhibited decreased high‐gamma power) in the left thalamus from 0 to 100 msec and from 200 to 300 msec in the nontarget condition. Unexpectedly, Group C showed the greatest increase in high‐gamma power in the left MFG from 300 to 400 msec in the nontarget condition. None of them showed a significant partial correlation with the change in cognitive composite scores.

**Figure 3:**
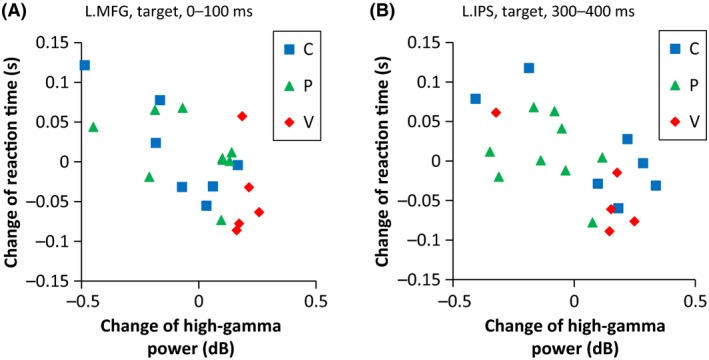
Scatter plots of the partial correlations. (A) Scatter plots between reaction time and change in high‐gamma power in the left middle frontal gyrus from 0 to 100 msec and (B) change in high‐gamma power in the left intraparietal sulcus from 300 to 400 msec in the target condition. The high‐gamma power of the preperiod, sex, and age were included in the model as covariates. False discovery rate multiple comparison correction (*q* < .05) was applied. Note that there are two points almost overlapping around (*x* = 0.1, *y* = 0) in Group P in Figure 3 (A).

The partial correlation analyses revealed significant correlations between the changes in reaction time and high‐gamma power in the left MFG from 0 to 100 msec (*r *=* *−0.65, *P *=* *0.004) and in the left IPS from 300 to 400 msec (*r *=* *−0.62, *P *=* *0.007), both of which survived after false discovery rate multiple comparison corrections. The scatter plots are presented in Figure 3. Again, none of them showed a significant partial correlation with the change in cognitive composite scores.

## Discussion

We found that the high‐gamma power changes in the left MFG from 0 to 100 msec and in the left IPS from 300 to 400 msec were associated with a change in behavioral performance (Fig. 3). Somewhat similar patterns in the two scatter plots suggest that their contribution to reaction time might not be independent, possibly because together they make up a frontoparietal network (Szczepanski et al. 2013), although the partial correlation between high‐gamma power in the left MFG from 0 to 100 msec and in the left IPS from 300 to 400 msec did not reach significance (*r *=* *0.35, *P *=* *0.123). The fact that these correlations were observed regardless of group suggests that high‐gamma power was sensitive to change in behavioral performance, not only induced by the effective cognitive training but also by less effective training and for other reasons, such as age‐induced changes due to the 2‐month intervention period. However, we also found that Group V showed increased high‐gamma power during target processing in the left MFG from 100 to 200 msec. Taken together, the increased high‐gamma power in the left MFG after intervention training in Group V very likely reflects a beneficial effect of on‐board cognitive training. The left MFG has been associated with top‐down attention (Gazzaley et al. 2007; Polich 2007). In line with this notion, these significant effects were observed in early time windows, suggesting that they were related to the executive function that plays an important role in top‐down anticipatory attention (Liang and Wang 2003). This interpretation seems reasonable given that the stimulus presentation was rhythmic (i.e., fixed stimulus presentation time and interstimulus interval) in this study.

Group P, who conducted similar cognitive training but with a PC, did not show this effect. This result was less expected in terms of the shared basic design of Group P's on‐PC training and Group V's on‐board training, but was consistent with previous findings showing that combined cognitive and physical training was more advantageous than cognitive or physical training alone (Bamidis et al. 2014). Indeed, Group V but not Group P showed significant improvements in cognitive functions as assessed by the neuropsychological test battery (Nozawa et al. 2015). Previous studies have shown that not only cardiovascular (aerobic) exercise but also coordinative exercise could improve cognitive functioning in older adults (Voelcker‐Rehage et al. 2010, 2011; Bherer et al. 2013; Voelcker‐Rehage and Niemann 2013). Motor coordination requires perceptual and cognitive processes, such as attention, that are important for mapping sensation to action (Voelcker‐Rehage and Niemann 2013). Thus, the observed difference might be due to a higher requirement for eye‐hand‐foot coordination in Group V. Another possible factor is motivation, which has been shown to contribute to training success (Dörrenbächer et al. 2014; Jaeggi et al. 2014). Although our participants seemed to be intrinsically motivated, as they voluntarily participated in the demanding intervention program, additional incentive might be provided by the training environment in a vehicle in Group V.

We also observed significant effects of intervention group in the left MFG and left thalamus in the infrequent nontarget condition. Unfortunately, we could not conclude whether these changes were associated with improved or declined cognitive performance due to the lack of a behavioral index (i.e., reaction time) in this condition. However, the similar patterns in Groups P and V, which were different from Group C, suggest that the changes reflect the difference in training tasks. Specifically, the nature of the cognitive training in Groups P and V was oriented toward the external environmental (i.e., stimulus‐driven), whereas the training for Group C was oriented inwardly (i.e., memory retrieval). Given that the thalamus plays an important role in filtering irrelevant but salient visual distractor (Strumpf et al. 2013), the high‐gamma power increase for nontarget stimuli in the left thalamus in Groups V and P might reflect an enhanced neural response to the salient external event, whereas the decrease in Group C might reflect a suppressed neural response to the same event. The fact that these significant effects were observed in early time windows is also consistent with this notion. Converging evidence suggests that MEG succeeded in measuring the signal from deep brain regions (Kimura et al. 2008; Parkkonen et al. 2009), even gamma‐band signals (Luo et al. 2007). Although the limited number of trials in this study needs careful consideration, there seemed to be a certain level of reliability because similar results were observed in a different sample with the same experimental settings as in our previous studies (Akimoto et al. 2013, 2014). On the other hand, the high‐gamma power increase in the left MFG from 300 to 400 msec in Group C for nontarget stimuli was not expected. One possible interpretation is that this reflects a far‐transfer effect of crossword puzzle training, by which working memory was marginally improved. In Group C, we observed a decreased neural response to the nontarget stimuli in the left thalamus, which possibly reflects early selective gating of goal‐irrelevant stimuli. Nevertheless, their task performance (i.e., task accuracy) was maintained, suggesting that compensatory mechanisms may be provided by alternative neural resources. It has been proposed that prefrontal regions play an important role in the compensatory mechanisms for maintained performance in senior adults (van Dinteren et al. 2014). A previous study further reported that the left MFG is the source of working memory capacity for suppressing goal‐irrelevant information in delay distractor filtering (Minamoto et al. 2010), which might rely on a different mechanism from that of early distractor filtering (McNab and Dolan 2014). Therefore, neuroplasticity in the left MFG may underlie mechanisms of the far‐transfer effect possibly occurring in Group C. This could also explain why the observed time window of the high‐gamma power change in the left MFG in Group C differed from those of the left thalamus in Groups V and P. In the standard condition, we observed no difference. This suggested that the effects of our interventions were more associated with cognitive processes rather than with simple perceptual processes. The lack of a significant effect of training group on behavioral performance might be due to a ceiling effect, as shown by the good performance in the elderly adult subjects in this study, which was comparable to young adult subjects in previous study (Akimoto et al. 2013).

In general, fast oscillations such as gamma are considered to reflect local interactions, whereas slow oscillations like delta or theta are considered to reflect long‐range interactions (Donner and Siegel 2011). In line with this idea, we found that cognitive training increased high‐gamma power in the regions specifically associated with oddball processing (i.e., attention, executive function, and working memory). A recent intervention study with elderly subjects at risk for dementia (Styliadis et al. 2015) reported that a combined cognitive and physical training regimen decreased resting state EEG activity in delta, theta, and beta bands in the precuneus/posterior cingulate cortex, which is a functional core of the default‐mode network (Fransson and Marrelec 2008; Utevsky et al. 2014). These results suggests that cognitive training with physical activity can induce positive neuroplastic changes in elderly people at both local and long‐range cortical interaction levels. Given that the phases of slow oscillations modulate the amplitude of fast oscillations (Canolty and Knight 2010), these changes may not be independent, but rather be complementary measures for the evaluation of training efficacy. For example, evaluating training effects during the cognitive task has the benefit of precise interpretation of the results, as the observed brain activity should be related to the task, and the behavioral index is often available. Similarly, evaluating the training effects from the analysis of resting state data is advantageous because doing so is feasible even when the subjects could not adequately perform that cognitive task. In addition, fast gamma oscillations during cognitive tasks would be more useful for evaluation of neuroplasticity in specific brain areas, whereas slow oscillations obtained from resting states would be more suitable for evaluation of neuroplasticity in the functional brain network among distant brain regions. It is also possible that the former may be more associated with the near‐transfer effect and the latter may be more related to the far‐transfer effect, although this conclusion remains largely speculative and further investigation is necessary to confirm it.

The major limitation of this study was a small and unbalanced number of subjects in each training group. Given the large inter‐ and intra‐individual variance among elderly subjects, a larger sample is needed to obtain stable results. This might be the reason why the high‐gamma power change did not significantly correlate with the changes in cognitive composite scores. Thus, our results should be considered preliminary, although the robust correlation between changes in high‐gamma power and changes in reaction time obtained from the same task is promising. Our small sample size was partly due to the 2011 Japan earthquake, but also due to the large number of excluded subjects in the analysis. The large number of exclusions was mainly caused by the following two reasons: (1) large magnetic noise due to metallic dental implants, and (2) brain atrophy due to age, the former seems to be specific problem when using electrophysiological measures as an index of the effectiveness of cognitive training in elderly subjects. Another limitation was that we did not include a passive control group, making our interpretation of the training effects somewhat speculative. Adding a passive control group would be helpful to distinguish the effect of cognitive training from an age‐induced effect caused by the intervention period. However, the addition of only a passive control group would confound the effect of participation in the intervention itself.

## Conclusions

We examined the effects of cognitive training in elderly people on high‐gamma activities and the relationship between the changes in high‐gamma power and behavioral performance. The results showed that high‐gamma power measurements changed differently among the training groups. In particular, Group V exhibited increased high‐gamma power during top‐down anticipatory target processing in the left MFG. The change in high‐gamma power in the left MFG was also associated with change in behavioral performance. Our preliminary findings suggested that changes in high‐gamma activities in executive control‐related regions are one of the neural mechanisms underlying training effects, and to the best of our knowledge, we are the first to suggest the usefulness of high‐gamma activities as an index of the effectiveness of cognitive training in elderly subjects.

## Conflict of Interest

T. Sunda, T. Shimizu, E. Tozuka, S. Hirose, and T. Nanbu are employed by Nissan Motor Co. Ltd., Japan. They have no other competing interests. All other authors have declared that no competing interests exist.
